# Zero new HIV infections: Mission impossible with current perceptions of young adults in Vhembe District regarding condom use

**DOI:** 10.4102/phcfm.v8i2.920

**Published:** 2016-05-25

**Authors:** Azwihangwisi H. Mavhandu-Mudzusi

**Affiliations:** 1 Department of Health Studies, University of South Africa, South Africa

## Abstract

**Background:**

Millennium Development Goal (MDG) number 6, target 6A focuses on halting and reversing the spread of HIV by 2015. South Africa is amongst the 189 countries that are involved in trying to meet the MDGs. In order to try and achieve this goal, South Africa, through its HIV and AIDS, STI and TB strategic plans has adopted the United Nations vision of ‘Zeros’, which include zero new HIV infections by the year 2015. One of the key interventions for achieving this is improvement in access to condoms.

**Aim:**

This article reports on perceptions of Vhembe District young adults regarding condom use.

**Setting:**

This study was conducted in one of the rural clinics in Vhembe District of the Limpopo Province: South Africa.

**Methods:**

A quantitative, cross-sectional design approach was followed. Data were collected using structured questionnaires administered to 372 young adults who came for healthcare services at one of the clinics in Vhembe District.

**Results:**

The findings indicated that there is a relationship between positive perceptions toward condom use and actual condom use. People who have positive attitudes toward condoms are also more likely to use them. In contrast, people with a negative attitude towards condoms are less likely to use them during sexual intercourse. Negative attitudes of health care providers were found to be a barrier that prevents sexually active individuals to access condoms in primary health care facilities.

**Conclusion:**

With current perceptions towards condom use, Zero new HIV infection will never be achieved. The researcher recommends development of strategies for promoting access and correct use of condoms.

## Introduction

Sub-Saharan Africa has the highest number of HIV and AIDS cases in the world.^1^ This region, which makes up 9% of the world population, carries two-thirds of the total global HIV cases.^1^ Thus, most countries in the sub-Saharan African region have generalised HIV epidemics with a prevalence exceeding 14%.^2^ Taking Zimbabwe and South Africa as examples, these states have HIV prevalence of 14.3 and 18%, respectively.^3^ Approximately 85% of such proportions of HIV infections are attributable to heterosexual sexual intercourse in the general population.^3,4^ In other words, HIV is spread primarily in sub-Saharan Africa through heterosexual contact. Whilst this is the case, it is worth noting that approximately 70% of new HIV infections in this region of Africa occur within heterosexual sero discordant relationships.^5^

South Africa is reported to have the highest number of people living with HIV in the world, with about 6.4 million of its inhabitants living with this infection.^6^ Taking this into account, South Africa is perceived by its Ministry of Health and WHO to have a generalised hyper-endemic HIV epidemic.^3^ In order to curb the spread of HIV, South Africa has adopted the United Nations vision of ‘Zeros’ that include zero new HIV infections by the year 2015.^7^ One of the key interventions for achieving this is improvement in access to condoms. Condoms are considered one of the most effective ways of preventing sexual transmission or contraction of HIV. The South African government, specifically its Health Department made condoms readily available for use at the health facilities.

However, condom use in Africa has proved to be an enormous challenge to health care workers and counsellors throughout this continent.^8^ Like other aspects of human sexuality, there are a number of myths surrounding the use of condoms. A study conducted by Kabikira^8^ in Uganda on the attitude of sexual practices in relation to the human immunodeficiency virus (HIV) and acquired immune deficiency syndrome (AIDS) noted that there was a general lack of willingness amongst respondents to use condoms during sexual intercourse. According to Burgard et al.^9^ most young people in contemporary South Africa are at a high risk of being infected with HIV through unprotected heterosexual intercourse. In addition to this, about one-third of these young adults claimed to have experienced at least one episode of sexual intercourse with or without condoms before their 20th birthday. These outcomes do not only indicate low condom usage, but they are also consistent with findings from the study by Burgard et al.^10^ on condom usage amongst young adults in South Africa. Low condom usage can be attributed to limited knowledge of this contraceptive amongst adolescents. A study by Ehlers^11^ confirms this by stating that adolescent mothers studied did not perceive condoms as contraceptives, but rather as methods for preventing HIV infection.

A study by Oyedele et al.^12^ on the prevention of HIV amongst young adults in Soshanguve, South Africa, also supports the association of limited condom use with lack of, or limited, knowledge. Some adolescents in the study reported to have multiple sexual partners and unprotected sexual intercourse despite the availability of condoms. This was also asserted by the study conducted by one of the studies conducted by Mavhandu-Mudzusi et al.^13^ on condom use amongst students requesting for emergency contraceptives.

The involvement of young adults in risky sexual activities without using condoms increases the risk of pregnancy and also contracting or transmitting sexually transmitted infections, including HIV.^14^ Despite this, sexually transmitted infections and teenage pregnancies continue to rise amongst young adults. This is certainly a public health concern for the whole of the South African population including thegovernment.^15^ The researcher has also observed an increase in the number of teenage pregnancies and HIV infection amongst young adults in Vhembe District despite the availability of free condoms at the public and private hospitals and clinics, as well as at public places. Limited condom usage will hinder the achievement of zero new HIV infections, which is part of Millennium Development Goal (MDG) number 6. This has made the researcher inquisitive about what the perceptions of young adults of Vhembe District are towards condom usage and how this may affect attainment of Zero HIV infections, which is a key to the attainment of MDG number 6.

## Purpose of the study

The purpose of the study was to investigate the perceptions of young adults with regard to condom use in the Vhembe District in the Limpopo Province of South Africa in order to guide the formulation of strategies for promoting correct and consistent condom use amongst young adults. These strategies are envisaged to be a contribution to the reduction of new HIV infections, which is one of the objectives towards the achievement of MDG number 6.

## Objectives

The objectives of the study were:

- To assess sexual activities amongst young adults.
- To describe the perceptions of young adults regarding condom use.
- To identify strategies for promoting consistent condom use during any ‘risky’ sexual intercourse.

## Research methods and design

### Study design

This study used a quantitative cross-sectional design. This was chosen because it is an approach that enables researchers to describe variables and their relationships. In other words, descriptive cross-sectional design can be used to measure attributes and examine associations between them.^16^ The attributes in this study relate to the young adults’ perceptions of condom use. Generally, in cross-sectional studies, data are collected at a specified point in time.^16^ This was also the case in this study, as data were collected only at one point in time, which was February to March 2014.

### Research site

The study was conducted in one of the rural clinics at Vhembe District in the Limpopo Province of South Africa. The clinic used in this study is situated within a district comprising six villages and one farm. The clinic is close to a main road, making it easily accessible to participants.

### Study population and sampling strategy

The population of this study was made up of all young adults of the Vhembe District who visit the public health care facilities for healthcare services. At the moment, there are no data on condom use by young adults in the Vhembe District in the Limpopo Province of South Africa. However, Burgard et al.^10^ conducted a study on condom usage amongst young adults in South Africa. They noted that about 41% of young adults failed to use condoms during sexual intercourse. The sample size for this study was therefore calculated using the statistics from the study by Burgard et al.^10^ The characteristics of the Burgard et al.^10^ study population can be considered to be similar to the young adults of the Vhembe District of Limpopo. Based on the results of Burgard et al.^10^, the minimum required sample size for this study was 372 respondents as per the calculation below, a figure also consistent with that offered by statisticians following consultation:

n=(zα2)2p(1−p)z2=(1.96)20.41(1−0.41)0.052=372 [Eqn 1]

A non-probability sampling method was used to recruit respondents in this study.^17^ The sampling technique employed was convenience sampling.^18^ Respondents were selected at the convenience of the researcher, as they made scheduled and non-scheduled visits to the clinics. All young adults were given information about the study by data collectors, and those who expressed willingness to participate in the study were approached individually whilst in the waiting area and encouraged to complete a consent form. A total of 372 young adults agreed to participate in the study by completing a consent form. Only young adults who completed consent forms were interviewed.

### Data collection

The questionnaire was developed and pre-tested with 10respondents before the execution of the main data collection of the study.

Data were collected in the clinic where the young adults received their health care services. A structured method was used for data collection using a structured questionnaire.^19^ Data collectors who were comfortable with English and Tshivenda languages were identified and trained, not only for ensuring an understanding of the content of the tool, but also for ensuring consistency in its application. The need for maintaining confidentiality was also included in the training and was addressed by using nurses and counsellors as data collectors; these were persons who worked in the study site and knew the young adults. Data collectors distributed the questionnaires to respondents for completion. Respondents who had difficulties with reading and writing were assisted in completing the questionnaires by the trained data collectors.

### Data analysis

In order to manage the data, all the completed questionnaires were captured and organised in the database management system of SPSS version 19. The data were then cleaned to ensure that only valid responses to questions were present in the database; logic checks were also conducted. In order to make the data meaningful, descriptive statistics (such as frequencies, percentages) as well as inferential statistics were used to analyse and present the data.

## Validity and reliability of the study

For validity, steps described in Bryman^20^ were followed. Questions were developed based on the objectives of the studies, focusing mainly on perceptions, and behaviour related to condom uses to ensure content validity. The questions were written using simple, unambiguous terms in order to accommodate the respondents’ level of understanding and also that of the data collectors. The questionnaire was reviewed by a panel of experts in the fields of sexual risky behaviours and quantitative studies to ensure content, construct, criterion and face validity. A field test was performed, and the questions were adapted based on feedback of the field test and from the experts in the field. Data collectors were trained on the data collection process. Further emphasis was made in relation to the sample size^19,21^ to ensure generalisability. In this study, the sample was not randomly selected, but its size was determined using a significance level or error rate of 0.05 (95%), meaning there was 95% chance of obtaining the same or similar results if the study was repeated. Sufficient data were collected using the minimum sample size of 372. However, because of the sampling approach used-convenience sampling- the generalisability of the study findings should be viewed with caution.

To ensure reliability, this study used a structured questionnaire as a data collection method. Because reliability is a multi-component concept, the researcher needed to decide in advance on the aspects of reliability (internal consistency, stability, or equivalence) when selecting instruments for the study. In this study, all these aspects were critical. The questionnaire was pilot tested with 30 young women at another clinic in the same district. Data from the pilot group were analysed using SPSS. Some irrelevant questions were removed. The data collector and researcher administered the questionnaire a second time to 10 youths from the pilot site, and the Cronbach’s alpha was 0.6.

## Ethical considerations

Permission to conduct the study was first granted by the University of South Africa’s Postgraduate Research Ethics Committee. This was followed by permission to conduct the study from the Vhembe Health District. The researcher also requested verbal permission from the operational Manager of the study site. The researcher assured all the relevant authorities that confidentiality of respondents and the clinic would be respected at all relevant times throughout the study.

Respondents were informed that participation in the study was absolutely voluntary, and their right to not answer any part, or all, of the questions was respected. Study respondents were asked to give their written consent to participate in the study. The data collection commenced after respondents expressed their willingness to participate by signing consent forms. Respondents were informed that they could withdraw from the study at any time. Study respondents were assured that their responses would be kept confidential and that the findings of the study would not be linked to them. Data collection was conducted in a private room, and each study respondent was given a unique identification code, which was used during data entry; their names were not recorded. The researcher and data collectors of this study maintained professional ethical and scientific conduct throughout the study.

## Results

### Demographic characteristics

The total sample size was (*n* = 372). The age of the majority of the respondents in this sample (97%) ranged from 18 to 25 years. This means only a minority of respondents (3%) were over 25 years of age. Out of the 372 respondents, nearly two-thirds (63%) of the respondents were female and 37% male. Thus, the survey respondents were mainly females. In relation to marital status, the majority of the respondents (94%) were single, and only 5% were married. Approximately 64% of the respondents who were single had a partner, and 36% had no partner at all. The dominant religion amongst the survey respondents was Christianity (97%), a small number were Muslims (2%) and the rest (1%) did not belong to any specific religion. Concerning the number of children belonging to the respondents, 76% of them had no children during the period of this study, 16% had one child each, 6% had two and 1% had three children respectively.

Regarding sexual intercourse, most of the respondents (57%) reported that they had had sex before data collection, whilst 43% claimed they had never been sexually active. Respondents were also asked at what age they had first engaged in sexual intercourse. At least 47% of the respondents reported to have had their sexual debut when they were between the ages of 10 to 18, whilst 51% claimed to have had their first sexual encounter between the ages of 18 to 25. Eighty per cent of the respondents who had had sexual intercourse agreed to using condoms during their sexual debut, whilst 20% reported they had not used condoms.

### Perceptions of young adults towards condom use

Perceptions of condom use per age and sex: Table 1 shows the young adults’ perceptions of condom use in relation to sex. Out of the 372 respondents, 57% claimed to have had sexual intercourse before data collection. Whilst about 52% of these were males, 60% were females. The relationship or association between sexual intercourse and sex was not significant (*p* = 0.137). In relation to age at first sexual intercourse, about 63% of the respondents who had had their first sexual encounters at 10 to 18 years of age were males, and 38% were females. With regard to the respondents who had had sexual intercourse at 18 to 25 years of age, 60% were noted as females and 32% were males. It is critical to note that a significant relationship was observed between age at first sexual intercourse with age and sex (*p* = 0.001).

**TABLE 1:** Perceptions of condom use per age and sex.

Variables	Category	*N* (%)	Male (%)	Female (%)	*p*
Ever had sex	Yes	212 (57)	52.20	60.10	0.137
Age of sexual debut	< 10 years	2 (1)	2.80	0.00	0.001
	10–18 years	97 (47)	63.40	38.00	-
	18–25 years	106 (51)	32.40	60.60	-
	25+ years	3 (1)	1.40	1.50	-
Perception of condom use, amongst sexually active	Good for one’s health	170 (80)	86.10	77.10	0.086
	Bad for one’s health	8 (4)	0.00	5.70	-
	No effect	34 (16)	13.90	17.10	-
Partner besides regular partner	Yes	46	33.30	17.80	0.039
Frequency of condom use	Always	102	61.40	42.40	0.01
	Sometimes	87	35.70	44.60	-
	Never	20	2.90	12.90	-
Negotiation of condom use	Yes	167	88.20	80.00	0.137

*Source*: Authors own work

The perceptions of respondents regarding condom use were explored. Although the relationship between perceptions of condom use and sex was not significant (*p* = 0.086), at least 170 (46%) respondents reported that using condoms is good for one’s health. Others, 34 (9%) claimed that using condoms would not have any impact on their health.

Another aspect of perception looked at during data collection was the frequency of condom use. At least 107 respondents (29%) claimed to always use a condom during sexual intercourse, and 61% of these were males and 42% were females. A significant relationship was found between frequency of condom use and sex (*p* = 0.01). This means that condom use was more prevalent amongst male than female study participants. Of the 87 (23%) respondents who reported to sometimes use condoms, about 36% were males and 46% were females.

Respondents who were not married were asked whether they had more than one sexual partner. Thirty three per cent of the 46 respondents who reported that they had more than sexual partner were males, 18% were females. This means the proportion of respondents with more than one partner was higher amongst males than females. A significant difference between the sexes (males and females) and number of sexual partners was noted in this study (*p* = 0.039).

**FIGURE 1:**
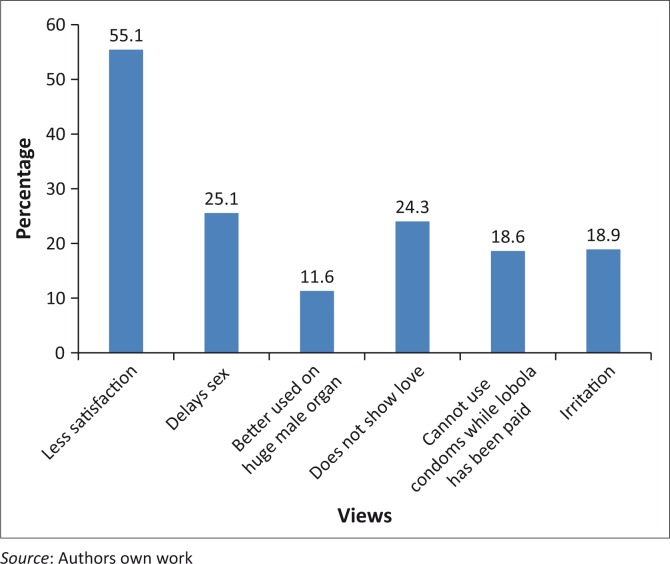
Views in relation to condom use.

Figure 1 shows the views of respondents regarding condom use. Most of the respondents (55%) claimed that using condoms during sexual intercourse offered less satisfaction, whilst 25% still believed that condom use delays sexual intercourse and ejaculation, and 24.3% reported that using a condom is tantamount to not showing affection for one’s partner. Only 19% of the respondents claimed that condoms cause irritation and may, therefore, deter their use.

### Reasons for inconsistent condom use

**FIGURE 2:**
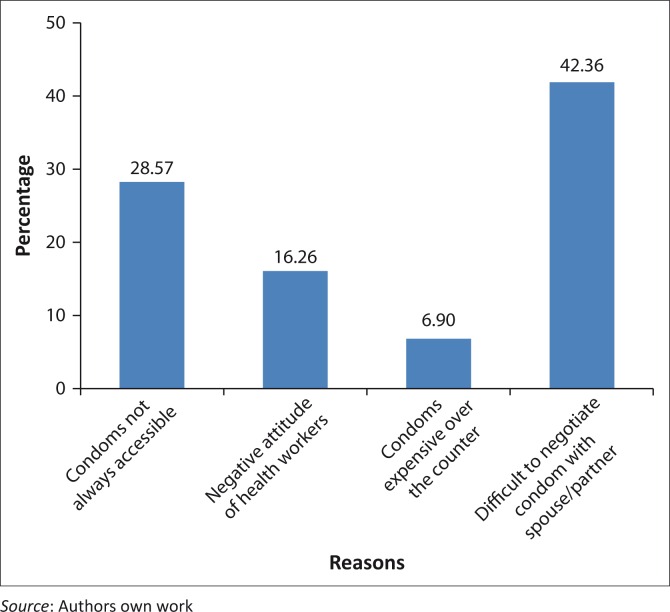
Reasons for inconsistent condom use.

Figure 2 indicates the reasons why respondents were not consistently using condoms. About 43% of respondents attributed their inconsistent use of condoms to the difficulty of negotiating with partners, 29% attributed it to problems with accessing condoms, whilst others (7%) attributed it to the cost of condoms.

### Strategies to promote consistent use of condoms

This section addresses aspects such as accessibility to condom use, strategies to improve the attitudes of health workers and strategies to promote correct and consistent use of condoms.

### Improving accessibility to condom use

Respondents were given three options to choose for improving accessibility. Most of the respondents (79%) agreed that the distribution of condoms to public places would enhance condom use, and 76% claimed that making condoms available at schools would also promote use. Approximately 68% of respondents believed that condom use can be promoted by using peer educators to distribute them.

### Strategies to improve the attitude of health workers

Participants have different views concerning approaches for improving attitudes of health workers toward condoms and their uses. Whilst about 88% reported that training would address health workers’ negative attitudes, some claimed that an effective management of complaints (72%) and the meaningful involvement of stakeholders (64%) would help with attitude change.

### Strategies to promote correct and consistent use of condoms

Several strategies for promoting correct and consistent use of condoms were raised. The use of several modes of media was considered by most respondents (57%) as an effective strategy for enhancing correct and consistent use of condoms amongst young adults. Sex education at schools was the second most preferred strategy as about 41% of respondents claimed that it would promote condom use. Involvement of peer educators and partnerships with stakeholders were the least preferred methods for promoting correct and consistent condom use.

## Discussion

### Demographic characteristics

A closer look at the demographic data revealed that more female young adults than males participated in this study. This disproportionate sex distribution of respondents might have been influenced by the sampling approach (convenient) used in the study. In addition to this, the disproportionate sex distribution of respondents could also be a reflection of the population of the study site district that the selected clinic serves, and the mix of the population of youths who attended this clinic for healthcare services. It is critical to state that the vast majority of young adults who attended the clinic were single and between the ages of 18 to 25. Young adults of this age group are considered to be highly active sexually, a view acknowledged by Burgard et al.^9^ Acknowledging this, it is not surprising to note this trend in this study as a high proportion of young adults had multiple sexual partners, had engaged in sexual intercourse and were mainly heterosexual before data collection.

The involvement in multiple sexual relationships has implications for unwanted pregnancies (with the possibility of mother to child transmission of HIV) and sexually transmitted infections, including HIV. An increasing in HIV hinders the achievement of zero new HIV infections, one of the objectives of achieving MDG 6. Burgard et al.^9^ agree with this by stating that most young people in contemporary South Africa are at a high risk of being infected with HIV through heterosexual sexual intercourse. The risks of unwanted pregnancy and the transmission of HIV are particularly high when young adults engage in unprotected sexual intercourse. Again, Burgard et al.^9^ support this assertion and state that about one-third of these young adults studied experienced at least one episode of pregnancy before their 20th birthday. According to Burgard et al.,^10^ this outcome indicates low condom usage.

In addition to education, religious beliefs could also influence young adults’ condom use. For example, Catholicism tends to discourage condom use in contrast to Islam and other forms of Christianity.^13^ The vast majority of respondents of this study were Christians. The study failed to differentiate or allocate respondents to the different forms of Christianity. Even though this is the case, it is noted in this study that only 29% of respondents (young adults) who are sexually active have used condoms consistently and correctly, whilst 71% have never used condoms or used them inconsistently. The young adults who failed to use condoms might be Catholics. This assertion is influenced by the view that Catholicism perceives the use of condoms as a promoter of sexual promiscuity that in turn may contribute to the spread of sexually transmitted diseases or infections, including HIV.^22^ Thus, in order to promote condom use, religious leaders should be involved in health promotion programmes to address some of the perceptions people have against condoms and their use.

### Sexual activity per age and sex

A high proportion (63%) of young adult males had their first sexual intercourse when they were 10 to 18 years old. It was noted in this study that most of the sexual activities of this age group were heterosexual in nature. Young adults, particularly those less than 15 years of age, might not be fully aware of the implications of engaging in heterosexual sexual activity. Examples of these implications include ability to negotiate condom use, and risks of sexual transmission of infections and unwanted pregnancies. In relation to female young adults, 60% experienced their sexual debut when 18 to 25 years of age. This delay in sexual intercourse by females should be interpreted with caution, as females may experience difficulties disclosing their sexual activities at an early age^15^ and this could be because of social acceptability reasons.

The outcomes of this study revealed that males were more likely than females to have more than one sexual partner. Relationships with multiple sexual partners were reported in the literature to be negatively associated with condom use.^13^ Although this is not clearly revealed in this study, it must be highlighted that inconsistencies in condom use amongst young adults were noted. In other words, young adults did not always use condoms during sexual intercourse. Undoubtedly, failure to use condoms may perpetuate the risk of transmission of HIV infections in the community, particularly in instances where multiple partners are involved. This will negatively affect the achievement of MDG 6.

### Young adults’ perceptions of condom use

It is consistently noted in the literature that people’s behaviour is sometimes influenced by their perceptions.^23^ Arguably, young adults with a negative perception of condoms are less likely to use them during sexual intercourse. The majority of the respondents of this study claimed that condoms prevent them from showing affection to their partners as well as preventing them from fully enjoying sexual intercourse. Acknowledging this, it is therefore not surprising to note that some of the young adults failed to use condoms during sexual intercourse. This suggests that young adults with a positive attitude or perception that condoms are good for their health are more likely to use the same during sexual encounters. The converse is true, as revealed in the study by Beltzer et al.^15^ of young adults in France who did not believe that condoms would protect them from contracting HIV. Such beliefs are dangerous by enabling these young adults to engage in unprotected sexual intercourse, whilst putting them at very high risk of contracting HIV. This indicates the need to educate young adults about safer sex.

### Strategies to promote condom use

The respondents of this study identified a number of approaches, or strategies, for condom enhancement. Most of the respondents suggested that improving accessibility would enhance the use of condoms amongst young adults. This means that condoms should be made available in public places and schools. This is in line with the recommendation by the South African National AIDS Council.^7^ In addition to this, there is a need for training and education about the benefits of condoms. Respondents claimed that the use of mass media such as television, newspapers and chatting sites would help to enhance condom use. Health promoters would use these forums to educate young adults about the benefits of condom usage as a contraceptive as well as a tool to prevent transmission of infections.

## Recommendations

The researcher recommends the development of strategies to improve access and utilisation of condoms in order to curb the spread of HIV infection, thus attempting to achieve zero new HIV infections, which is one of the key strategies for achieving MDG 6 The following are some of the focus areas to be included in the strategy:

- Re-training of health care providers to address a negative attitude is a matter of priority.
- Using social media as an avenue to communicate consistent and correct condom use.
- Reviewing of the current curriculum so that Life Orientation as a school subject would include all aspects of sexuality, sex and the benefits of condom use.
- Intensifying condom distribution at public places and making condoms available at schools to improve accessibility is important. Peer educators and community health care workers can be used to distribute condoms to local schools and hot spots in communities.
- Empowering of women to negotiate condom use with their sexual partners.

## Conclusion

The study indicated that there is a relationship between perceptions towards condom use and the actual usage of condoms. It means that young adults, who have a positive attitude towards condoms, are also more likely to use them, whereas young adults with a negative attitude towards condoms are less likely to use condoms during sexual intercourse. The findings also indicated that males use condoms more than females, and females are not able to negotiate condom use with their partners. Negative attitudes of health care providers were found to be a barrier that prevented sexually active individuals from accessing condoms in Primary Health Care facilities. This difficulty in accessing condoms increases risk involvement in risky sexual behaviours that fuel the spread of HIV infections. This continuous spread of HIV infections opposes the required objective of achieving zero new HIV infections. As long as the zero new HIV infections is not reached, it will be difficult, if not impossible, to achieve MDG 6.

### Limitations of the study

Convenience sampling that was used in this study means that the findings of this study can only be used for the population from which the sample was drawn, and they cannot be generalised to all the young adults of the primary healthcare facilities in the Limpopo Province, or even to the rest of South Africa. It is, however, important to note that most of the findings are in agreement with findings that have been made elsewhere, as discussed. The findings can therefore be adopted in other urban settings similar to the setting in which the study was conducted. The study adopted a cross-sectional descriptive methodology. This means data collection was carried out at one point in time, which was at one clinic in the Vhembe District. Adopting a longitudinal approach to data collection would have enhanced insight into this area of study, as it may have allowed for more persistent views of respondents to be revealed, thereby improving the validity and reliability of the data.
